# Retraction: BMP9 inhibits proliferation and metastasis of HER2-positive SK-BR-3 breast cancer cells through ERK1/2 and PI3K/AKT pathways

**DOI:** 10.1371/journal.pone.0320173

**Published:** 2025-03-03

**Authors:** The PLOS One Editors

After this article [1] was published, concerns were raised regarding results presented in Figs 3–5.

Specifically:

- The following lanes appear similar despite representing different experimental conditions:
  ◦ Fig 3C ERK1/2 panel lanes 1–4 and Fig 4A ERK1/2 panel lanes 2–5
- In Fig 4A, the B-actin panel appears similar to the Fig 4B B-actin panel, despite representing different experimental conditions
- There appear to be vertical and horizontal discontinuities in the following panels when levels are adjusted to visualize the background:
  ◦ Fig 4A p-ERK1/2 between lanes 3 and 4
  ◦ Fig 4A ERK1/2 between lanes 5 and 6
  ◦ Fig 4A and Fig 4B B-actin panels, above lanes 2 and 3
- In Fig 4B p-AKT panel, when levels are adjusted to visualize the background, there appears to be a region of the background signal around lanes 4 and 5 that does not match the surrounding areas
- In Fig 5D, the following panels appear to partially overlap:
  ◦ p-ERK1/2 BMP9 and p-AKT BMP9 panels
  ◦ HER2 Blank panel and p-HER2 Blank panel rotated 180°
  ◦ The inset of p-HER2 siBMP9 panel and HER2 siBMP9 panel

The author YL stated that errors were made during the preparation of Figs 3-5, but they stated that the uncropped underlying image data for the results presented in these figures is no longer available due to the age of the experiments. In the absence of the underlying data, the image concerns cannot be fully resolved.

In light of the above unresolved concerns that question the integrity and reliability of the reported results and conclusions, the PLOS One Editors retract this article.

The author YL notified the journal that all authors did not agree with the retraction. The other authors either did not respond directly or could not be reached.
